# Metagenomics-Metabolomics Reveals the Alleviation of Indole-3-Ethanol on Radiation-Induced Enteritis in Mice

**DOI:** 10.4014/jmb.2502.02037

**Published:** 2025-07-18

**Authors:** Hua Zhong, Yipeng Song, Shanliang Hu, Chengxu Zhang, Lili Li

**Affiliations:** 1 School of Pharmacy, Binzhou Medical University, Yantai 264003, P.R. China; 2 Yantai Institute of Coastal Zone Research, Chinese Academy of Sciences, Yantai 264003, P.R. China; 3 Department of Radiotherapy, Yantai Yuhuangding Hospital, Yantai 264000, P.R. China

**Keywords:** Gut microbiota, metabolites, radiation enteritis, radiotherapy, indole-3-ethanol

## Abstract

Indole-3-ethanol (IEt), a small molecule metabolite from intestinal microbial tryptophan metabolism, has been established to have anti-inflammatory properties. However, its effect on radiation-induced enteritis has not been reported. Here, we aim to explore the effects and potential mechanisms of IEt on radiation enteritis. C57BL/6J mice were orally administered an IEt solution before radiation exposure. Inflammatory factors, including IL-17A, IFN-γ, IL-6 and IL-1β, were detected using enzyme-linked immunosorbent assay. Colonic histopathology was assessed through H&E staining. Subsequently, gut microbiota and its metabolites were analyzed using metagenomics and metabolomics. The results suggested that IEt alleviated radiation-induced enteritis, as evidenced by improved colonic structural integrity, decreased levels of pro-inflammatory factors like IL-17A, and the restoration of intestinal microecological and metabolic balance. IEt enriched the abundance of Lachnospiraceae family members, particularly the genus *Roseburia* – a known anti-inflammatory commensal. In addition, IEt upregulated the levels of metabolites with anti-inflammatory effects such as indole-3-carbinol, pteridine, and pyropheophorbide-a. Furthermore, *Roseburia* was significantly positively correlated with indole-3-carbinol and negatively correlated with the pro-inflammatory factor IL-17A. Therefore, IEt may alleviate radiation enteritis through *Roseburia*-indole-3-carbinol and *Roseburia*-IL-17A axes. This study revealed the potential mechanisms by which IEt alleviated radiation enteritis, providing a potential protective candidate for radiation enteritis.

## Introduction

Cancer remains a major global health burden, with the International Agency for Research on Cancer estimating approximately 19.974 million new cancer cases worldwide in 2022 [1]. Radiotherapy, a cornerstone of cancer therapy, is utilized by over 50% of patients [2, 3]. However, when applied to pelvic tumors, radiation exposure often affects adjacent intestinal tissues due to their anatomical proximity, significantly increasing the risk of radiation enteritis (RE) [4]. Acute RE manifests in 60%–80% of patients within three months post-treatment, while 5–20%may progress to chronic cases [5, 6]. For intra-abdominal and pelvic malignancies, the irradiated field is often close to the intestine, resulting in radiation-induced damage that disrupts intestinal microecology and can lead to radiation enteritis [7]. The occurrence of radiation enteritis can lead to weight loss and a compromised immune response in patients receiving radiotherapy, potentially leading to treatment interruptions [8].

Although the complete etiology of radiation enteritis is not fully understood, emerging evidence indicates that the gut microbiome plays a crucial role in the onset of this condition. Radiotherapy may alter the diversity of intestinal microbiota and reduce the abundance of beneficial bacteria [4]. These alterations in the microbial community may increase intestinal permeability, allowing substances like lipopolysaccharide that should not normally pass through the intestinal wall to enter the body, provoking an immune response and consequently heightening inflammation [9, 10]. Furthermore, the long-term surviving mice after radiation exhibited a unique gut microbiota enriched in the family Lachnospiraceae compared to the control group [11]. Fecal microbiota transplantation (FMT) experiments revealed that germ-free mice receiving irradiated donor microbiota developed more severe intestinal mucosal damage and higher inflammation scores than those receiving control microbiota [12]. Therefore, gut microbiota is crucial in the development of radiation enteritis.

In addition, gut microbiota-derived metabolites also play an important role in radiation enteritis. Indole-3-carboxaldehyde, a crucial tryptophan metabolite, has been shown to increase the levels of beneficial bacteria and stimulated the AhR/IL-10/Wnt pathway, thereby fostering the growth of intestinal epithelial cells in irradiated mice [13]. Moreover, another study reported that certain intestinal microbial metabolites such as short-chain fatty acids (SCFAs) and derivatives of tryptophan like indole-3-carboxaldehyde and kynurenic acid serve as radioprotective agents [11]. It has been reported that intestinal microbial metabolites such as indole-3-propionic acid, can mediate some effects induced by FMT, thereby playing a protective role against gastrointestinal toxicity [14]. These findings suggest that gut microbial metabolites protect against the adverse side effects of radiation exposure. Although microbial metabolites are known to modulate host physiology in health and disease [15, 16], the functional roles and mechanistic underpinnings of most gut-derived small molecules remain poorly characterized. Indole-3-ethanol (IEt), a small molecule metabolite produced by intestinal microorganisms, is also an intermediate in the tryptophan metabolic pathway. Its effects on radiation-induced enteritis have not been reported. Based on the close relationship between radiation enteritis and intestinal microecological disorder, this study explored the effects and potential mechanisms of IEt on radiation-induced enteritis in mice through integrated metagenomics and metabolomics analysis of gut microbiota, providing theoretical support for the prevention of radiation enteritis.

## Materials and Methods

### Animals and Experimental Design

**Fig. 1:**
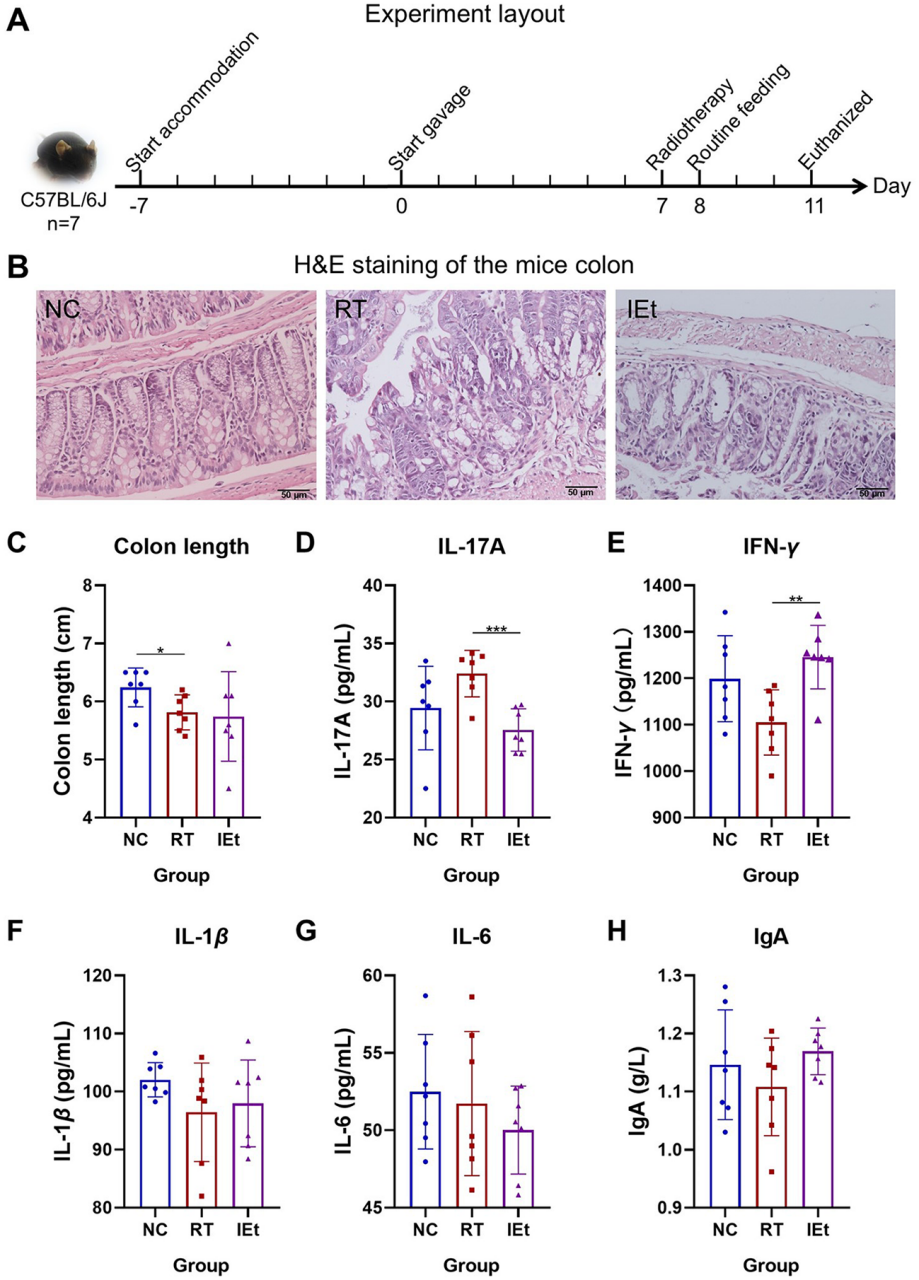
Effects of indole-3-ethanol on radiation enteritis. (**A**) Experiment layout. (**B**) The results of H&E staining of the mice colon, scale bar, 50 μm. (**C**) The length of colon. (**D-H**) The levels of (**D**) IL-17A, (**E**) IFN-γ, (**F**) IL-1β, (**G**) IL-6 and (**H**) IgA. *p < 0.05, **p < 0.01, ***p < 0.001. NC, the control group; RT, the radiotherapy group; IEt, IEt administration + radiotherapy group.

Six-week-old female C57BL/6J mice, obtained from Jinan Pengyue Experimental Animal Technology Co., Ltd.,(China), were housed in a specific pathogen free environment, maintained at an ambient temperature ranging from 22°C–25°C, with 50%–55% humidity, and a lighting cycle of 12 h of light followed by 12 h of darkness. The mice were provided with sterile water and standard chow (Research Diets D12450B, HFK Bioscience Co., Ltd., China). Table S1 detailed the components of the standard chow. After a 7-day acclimation phase (Fig. 1A), the mice were randomly assigned to three groups (*n* = 7): a non-treated control (NC) group, a radiotherapy (RT) group, and a group that received IEt supplementation along with radiotherapy (IEt). During the first 7 days, the IEt group received 600 mg/kg/day [17] of IEt (Shanghai Yuanye Bio-Technology Co., Ltd., China) via oral administration, dissolved in a vehicle solution of 20% DMSO and 80% water. Simultaneously, the other two groups received the same volume of the vehicle solution through gavage. On day eight, the RT and IEt groups were subjected to a single dose of high-intensity whole-abdomen radiation using 6 MV X-rays produced by medical linear accelerators (Elekta, Sweden). Radiation was delivered at a total dosage of 12 Gy at a rate of 300 MU/min, with a source-to-skin distance of 100 cm, and the radiation field covered the area from the xiphoid to the pubic symphysis [18]. The NC group did not receive radiation. Following the treatment, the mice were fed a common diet with water and chow freely available for 3 days before euthanasia. Animal experiments were approved by the Animal Ethics Association of Yantai Yuhuangding Hospital (Approval number 2023-372).

### Sample Collection

Upon completion of the experimental protocol, mice were euthanized via decapitation following anesthesia induced by intraperitoneal administration of pentobarbital sodium (50 mg/kg) [19]. Fecal samples were collected from mice prior to their euthanasia. Each mouse was housed in sterile cages, where the fecal samples were collected in sterile tubes, quickly frozen using liquid nitrogen, and stored at -80°C. These procedures were completed within a strict timeframe of 2 h. Blood samples were collected from the orbital sinus of the mice, combined with EDTA to prevent clotting, and centrifuged at 4°C, 3000 rpm for 10 min to separate the plasma. After dissection, the length of the colon was measured.

### Enzyme-Linked Immunosorbent Assay (ELISA)

The levels of IL-17A, TNF-α, IFN-γ, IL-1β, IL-6, and IgA in the plasma of mice were measured using ELISA kits obtained from mlbio (China), in accordance with the protocols provided by the manufacturer.

### Histopathological Analysis of the Colon in Mice

Following euthanasia, the colons were preserved in a 4% paraformaldehyde solution at 25°C. Afterward, 5 μm thick sections were prepared, embedded in paraffin, stained with hematoxylin and eosin (H&E) according to established protocols [20], and observed under an optical microscope (20×objective; Olympus Corp., Japan).

### Metabolome Analysis

A total of 100 mg of feces was individually ground in liquid nitrogen and then suspended in prechilled 80%methanol with vigorous vortexing. The samples were kept on ice for 5 min, followed by centrifugation at 15,000 g for 20 min at 4°C. A portion of the supernatant was diluted to 53% methanol using LC-MS grade water. The samples were then transferred to new Eppendorf tubes and centrifuged again under the same conditions. The supernatant was subsequently analyzed using LC-MS/MS [21]. UHPLC-MS/MS analyses were conducted with a Vanquish UHPLC system (Thermo Fisher Scientific, Germany) paired with either an Orbitrap Q Exactive HF (Thermo Fisher Scietific) at Novogene Co., Ltd., (China). The samples were injected onto a Hypersil Gold column (100 × 2.1 mm, 1.9 μm) employing a 12-min linear gradient at a flow rate of 0.2 ml/min. Metabolite identification was conducted against a high-quality secondary spectrum database based on adduct ions with a mass deviation of 10 ppm. Background ions were eliminated using blank sample data, and quantitative results were normalized to achieve relative peak areas. Ultimately, the results for metabolite identification and relative quantification were obtained. Data processing was performed on a Linux operating system (CentOS version 6.6), using R and Python. These metabolites were annotated using the KEGG database (https://www.genome.jp/kegg/pathway.html). We applied univariate analysis (*t*-test) to calculate the statistical significance (*p*-value).The metabolites with VIP>1, *p*-value<0.05, and fold change≥2 or FC≤0.5 were considered to be differential metabolites. Volcano plots were used to filter metabolites of interest based on log2 (FoldChange) and -log10 (*p*-value) of metabolites by ggplot2 in R language. The raw data of untargeted metabolomics were deposited in the National Microbiology Data Center (NMDC) with the accession number NMDC10019551.

### Metagenomic Analysis

DNA was isolated from mouse fecal samples using TIANamp Stool DNA Kit (TIANGEN Biotech Co., Ltd., China), adhering to the manufacturer's instructions. The genomic DNA was then randomly fragmented, and libraries were created for sequencing purposes. These fragments underwent end repair, A-tails addition, and the incorporation of adapters compatible with Illumina technology. PCR amplification was performed on the fragments that had been ligated with adapters, followed by size selection and purification. The quality and quantity of the constructed library were assessed utilizing Qubit and real-time PCR, while the analysis of fragment size distribution was performed using a bioanalyzer. After quantification, the libraries were pooled and prepared for sequencing on Illumina platforms (Novogene Co., Ltd.) in line with the required library concentration and data volume.

The Raw Data acquired from Illumina sequencing platforms underwent preprocessing using Readfq (https://github.com/cjfields/readfq) to produce Clean Data for subsequent analysis. The steps involved in preprocessing comprised: a) elimination of reads exhibiting a high proportion of low-quality bases (quality threshold ≤38, with a default length of 40 bp); b) removal of reads containing an excessive number of N bases; and c) eradication of reads demonstrating considerable adapter overlap. To mitigate potential host contamination, Clean Data were aligned with a reference genome database specific to the host to filter out reads likely originating from the host. This mapping was executed utilizing Bowtie2 software (http://bowtie-bio.sourceforge.net/bowtie2/index.shtml) with standard parameters: --end-to-end, --sensitive, -I 200, and -X 400 [22, 23].

The assembly process of the Clean Data utilizing the MEGAHIT software with the following parameters: --presets meta-large (--end-to-end, --sensitive, -I 200, -X 400) [22, 24]. The resulting Scaffolds were segmented at N junctions to create Scaftigs that did not contain N bases.

The raw data of metagenomic sequencing were stored in the NCBI Sequence Read Archive with the accession number PRJNA1165328.

### Statistical Analysis

Comparisons between groups were carried out using the t-test implemented in the SPSS program (version 24.0; SPSS Inc., USA). The findings are presented as mean ± standard deviation (SD), with a *p*-value below 0.05 considered statistically significant (*p < 0.05, **p < 0.01, ***p < 0.001).

## Results

### Effects of IEt on Colon Structure

H&E staining results demonstrated that the colon structure in the NC group was intact, exhibiting clear crypts. In contrast, the colon structure in the RT group displayed damage, characterized by concave morphology, reduced crypt numbers, and structural destruction of the crypts (Fig. 1B). The crypts were compromised after radiation, whereas IEt treatment effectively restored the crypt structure in the mice. Comparative analysis of colon length (Fig. 1C) revealed a significant decrease in the RT group compared to the NC group (*p* = 0.028). Additionally, no statistically significant differences in colonic length were observed when comparing the IEt group with either the RT or NC groups.

### Anti-Inflammatory Effects of IEt

To further investigate radiation enteritis, we assessed the levels of inflammatory markers, including IL-17A, IL-6, IFN-γ, IL-1β, and IgA, in the plasma of mice (Fig. 1D-1H). Notably, the t-test results showed that the level of IL-17A in the RT group increased by 10.8% compared to the NC group (*p* = 0.087), while the level of IL-17A in the IEt group significantly decreased by 14.98% compared to the RT group (*p* = 0.0005). The RT group exhibited reduced IFN-γ levels compared to the NC group, a difference that was alleviated by IEt intervention. Additionally, no significant changes were detected in the levels of IL-1β, IL-6, and IgA.

### IEt Improved the Microbiota Disorder

**Fig. 2:**
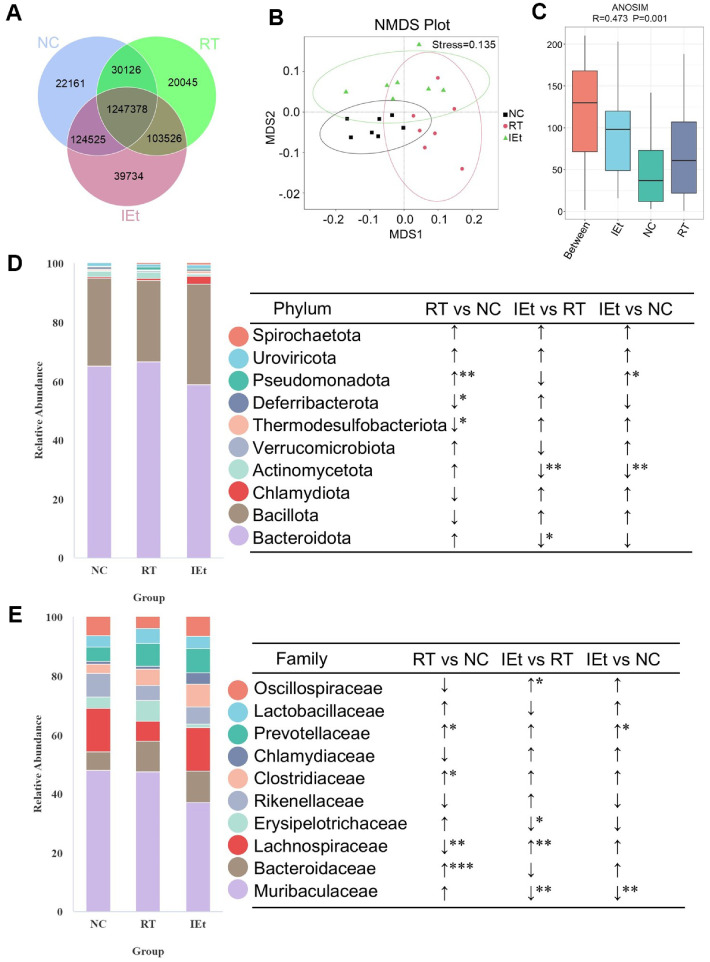
Gut microbiota composition and structure analysis. (**A**) Venn diagram. (**B**) Non-metric dimensional scaling (NMDS) plots of the gut microbiota on family level; (**C**) Analysis of similarities (ANOSIM) among the NC, RT and IEt groups based on the family level; (**D-E**) The relative abundance of the top ten (**D**) phyla and the top ten (**E**) families. *p < 0.05, **p < 0.01, ***p < 0.001. NC, the control group; RT, the radiotherapy group; IEt, IEt administration + radiotherapy group.

The Venn diagram illustrated the distribution of genes among the groups and highlighted the common genes shared (Fig. 2A). Subsequently, differences in the composition of intestinal microbiota across the three groups were assessed utilizing the non-metric dimensional scaling (NMDS) approach (Fig. 2B) and the analysis of similarities (ANOSIM) method based on Bray-Curtis distance at the family level (Fig. 2C). The results indicated significant inter-group variation in gut microbiota compared to intra-group differences (*p* < 0.05).

Evaluation of the intestinal microbiota at the phylum level revealed that Bacteroidota and *Bacillota* (*Firmicutes*) were the dominant phyla across all three study groups (Fig. 2D). After radiotherapy, a significant decline was observed in the prevalence of the Thermodesulfobacteriota and Deferribacterota phyla, while the Pseudomonadota phylum experienced a substantial increase in the RT group compared to the NC group. Furthermore, the IEt group exhibited significantly lower relative abundances of the Actinomycetota phylum and Bacteroidota when contrasted with the RT group.

Analysis of intestinal microbiota at the family level indicated that Muribaculaceae was the dominant microbial family across all three groups (Fig. 2E). After radiotherapy, the RT group exhibited a substantial increase in the prevalence of Bacteroidaceae, Clostridiaceae, and Prevotellaceae families, accompanied by a notable decrease in the Lachnospiraceae family (*p* = 0.0033) compared to the NC group. In the IEt group, in contrast to the RT group, there was a significant decline in the prevalence of Muribaculaceae and Erysipelotrichaceae families, while Lachnospiraceae (*p* = 0.0037) and Oscillospiraceae families experienced a significant increase.

### The Core Strain Affected by IEt Was Lachnospiraceae

**Fig. 3:**
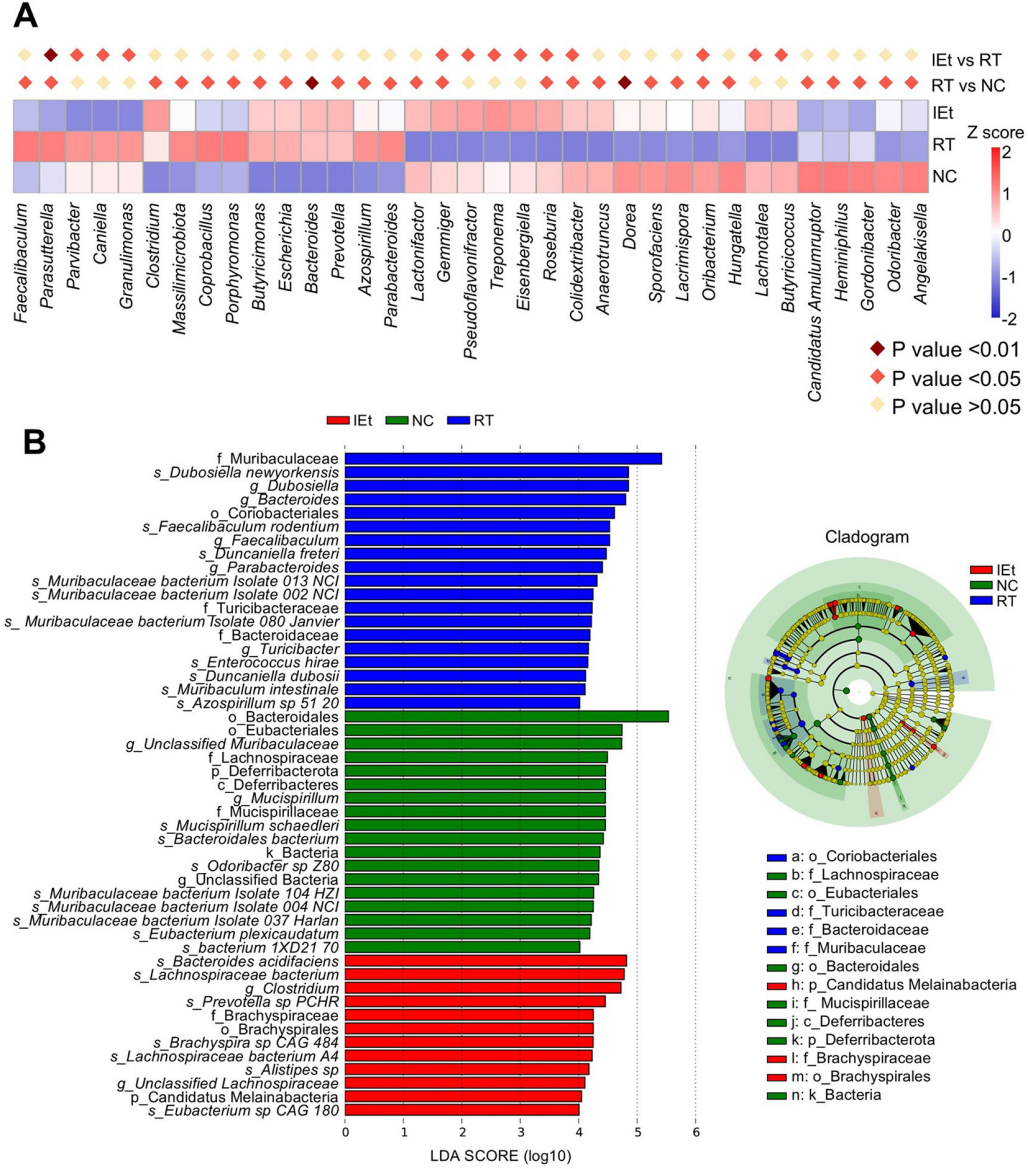
Alterations in gut microbiota across various taxonomic levels. (**A**) MetaStat analysis of the top thirty-five distinct genera across the three groups; (**B**) LEfSe analysis among the three groups. NC, the control group; RT, the radiotherapy group; IEt, IEt administration + radiotherapy group.

Analysis of MetaStat at the genus level (Fig. 3A) revealed a significant increase (*p* <0.05) in the relative abundance of *Parasutterella* following radiotherapy in contrast to the decrease (*p* < 0.01) observed after IEt treatment. Additionally, the RT group exhibited a notable reduction (*p* < 0.05) in the relative abundances of *Gemmiger* (*p* = 0.0476), *Roseburia* (*p* = 0.0344), *Colidextribacter* (*p* = 0.0241) and *Oribacterium* (*p* = 0.0265), whereas the IEt group experienced a marked increase (*p* < 0.05) in their abundance. Among the top 35 genera identified in the MetaStat analysis, a total of seven genera belonging to the family Lachnospiraceae were identified, namely *Eisenbergiella*, *Roseburia*, *Dorea*, *Sporofaciens*, *Lacrimispora*, *Oribacterium*, and *Lachnotalea*. Among these genera, *Roseburia* (*p* = 0.0344), *Dorea* (*p* = 0.0075), *Sporofaciens* (*p* = 0.0265), *Lacrimispora* (*p* = 0.0241) and *Oribacterium* (*p* = 0.0265) significantly decreased after radiotherapy, and *Eisenbergiella* (*p* = 0.0466) and *Roseburia* (*p* = 0.0363) significantly increased following IEt treatment. The abundance of the genus *Roseburia* decreased by 55.87% in the RT group compared to the NC group, and increased by 155.14% in the IEt group compared to the RT group.

LEfSe analysis (Fig. 3B) identified 49 distinct taxa across the three study groups (LDA score > 4). The NC group exhibited an enrichment of *Mucispirillum schaedleri*, *Bacteroidales bacterium*, *Eubacterium plexicaudatum*, and other bacterial species. After radiotherapy, there was an enrichment of *Dubosiella newyorkensis*, *Faecalibaculum rodentium*, *Duncaniella freteri*, and additional bacteria. After IEt supplementation, *Bacteroides acidifaciens*, *Lachnospiraceae bacterium*, and other species were enriched.

**Fig. 4:**
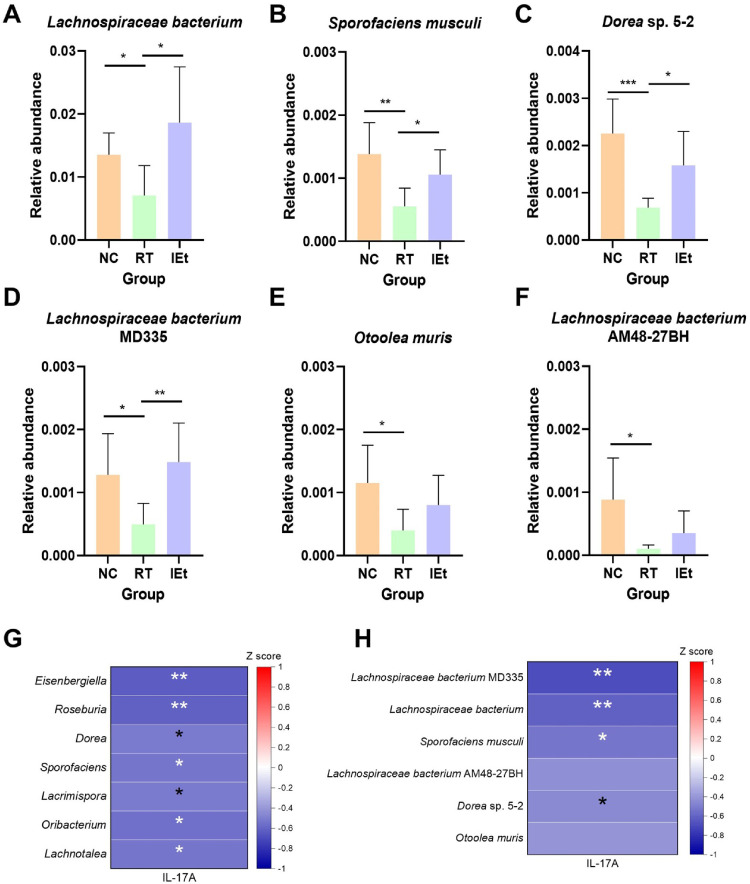
Significantly changed species of Lachnospiraceae and correlation analysis. (**A-F**) Significantly changed species of the family Lachnospiraceae among the three groups, (**A**) *Lachnospiraceae bacterium*; (**B**) *Sporofaciens musculi*; (**C**) *Dorea* sp. 5-2; (**D**) *Lachnospiraceae bacterium* MD335; (**E**) *Otoolea muris*; (**F**) *Lachnospiraceae bacterium* AM48-27BH; (**G-H**) Spearman correlation analysis between IL-17A level and (**G**) genera of the family Lachnospiraceae and (**H**) species of the family Lachnospiraceae. *p < 0.05, **p < 0.01, ***p < 0.001. NC, the control group; RT, the radiotherapy group; IEt, IEt administration + radiotherapy group.

Analysis of microbiota at the species level (Fig. S1) showed that, compared to the NC group, the abundances of several species, including *Lachnospiraceae bacterium*, *Lachnospiraceae bacterium* AM48-27BH, *Sporofaciens musculi*, *Hungatella hathewayi*, and *Dorea* sp. 5-2, were significantly altered post-radiotherapy, returning to baseline levels following IEt. Among the 35 most abundant species, six species belonging to the family Lachnospiraceae were noted: *Lachnospiraceae bacterium* MD335, *Lachnospiraceae bacterium*, *Sporofaciens musculi*, *Lachnospiraceae bacterium* AM48-27BH, *Dorea* sp. 5-2, and *Otoolea muris*, all of which exhibited significant changes in abundance (*p* < 0.05) after radiotherapy or IEt treatment. In addition, the t-test was conducted with a 95% confidence interval among the three groups (Fig. 4A-4F). The results showed that the abundance of species belonging to the Lachnospiraceae family, including *Lachnospiraceae bacterium* (*p* = 0.0140), *Lachnospiraceae bacterium* MD335 (*p* = 0.0200), *Sporofaciens musculi* (*p* = 0.0037), *Lachnospiraceae bacterium* AM48-27BH (*p* = 0.0210), *Otoolea muris* (*p* = 0.0170), and *Dorea* sp. 5-2 (*p* = 0.0009) were significantly decreased (*p* < 0.05) after radiotherapy. Among these, *Lachnospiraceae bacterium* (*p* = 0.0140), *Lachnospiraceae bacterium* MD335 (*p* = 0.0047), *Sporofaciens musculi* (*p* = 0.0190), and *Dorea* sp. 5-2 (*p* = 0.0920) significantly increased in the IEt group compared to the RT group.

### Correlations between Lachnospiraceae and IL-17A

Spearman correlation analysis (Fig. 4G-4H) revealed that, at the genus level, IL-17A exhibited significant negative correlations with genera from the Lachnospiraceae family, including *Eisenbergiella* (*p* = 0.0025), *Roseburia* (*p* = 0.0029), *Dorea* (*p* = 0.0190), *Sporofaciens* (*p* = 0.0150), *Lacrimispora* (*p* = 0.0180), *Oribacterium* (*p* = 0.0110), and *Lachnotalea* (*p* = 0.0120). At the species level, IL-17A was significantly negatively correlated with species from the Lachnospiraceae family, such as *Lachnospiraceae bacterium* MD335 (*p* = 0.0007), *Lachnospiriceae bacterium* (*p* = 0.0036), *Sporofaciens muculi* (*p* = 0.0150), and *Dorea* sp. 5-2 (*p* = 0.0440).

### Effects of IEt on Microbial Metabolic Functions

**Fig. 5:**
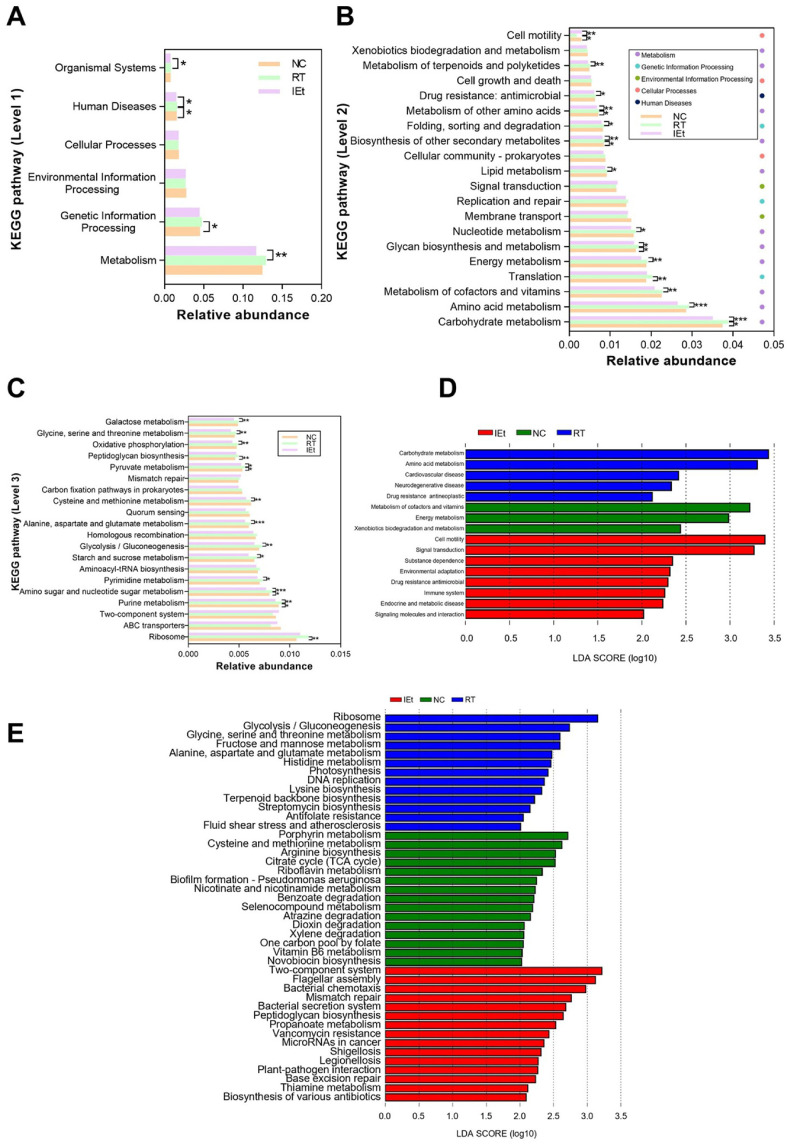
KEGG pathway analysis. (**A-C**) The relative abundance of KEGG pathway at (**A**) level 1, (**B**) level 2 and (**C**) level 3; (**D-E**) The difference of metabolic pathway abundance by LEfSe analysis (LDA score>2) at (**D**) level 2 and (**E**) level 3. *p < 0.05, **p < 0.01, ***p < 0.001. NC, the control group; RT, the radiotherapy group; IEt, IEt administration + radiotherapy group.

We analyzed the top 20 metabolic pathways of microbial metabolic functions (Fig. 5). Notably, metabolism exhibited the highest abundance at level 1 (Fig. 5A). Moreover, at level 2 (Fig. 5B), pathways such as carbohydrate metabolism, amino acid metabolism, and energy metabolism were particularly prominent. After radiotherapy, the abundance of metabolic pathways was significantly increased, including the biosynthesis of secondary metabolites, glycan biosynthesis and other amino acid metabolism, which were reversed by IEt. In contrast, the pathway of cell motility exhibited a significant decrease in abundance after radiotherapy, which was subsequently increased by IEt treatment. At level 3 (Fig. 5C), the abundance of amino sugar and nucleotide sugar metabolism, purine metabolism and pyruvate metabolism significantly increased in the RT group relative to the NC group, but these changes were reversed by IEt treatment.

LEfSe analysis at level 2 (Fig. 5D) revealed that the NC group exhibited enrichment in pathways such as metabolism of cofactors and vitamins, energy metabolism, and xenobiotics biodegradation. Additionally, the RT group showed enrichment in pathways related to cardiovascular disease, neurodegenerative disease, and drug resistance antineoplastic, while the IEt group displayed enrichment in pathways related to drug resistance antimicrobial, immune system, endocrine and metabolic disease. An in-depth examination at level 3 (Fig. 5E) using LEfSe revealed the enrichment of various metabolic pathways in all three groups. Notably, the NC group demonstrated enrichment in metabolic processes associated with porphyrin metabolism, arginine biosynthesis, citrate cycle (TCA cycle), and other entries. The RT group exhibited enrichment in pathways related to ribosome, glycolysis/gluconeogenesis, and other entries. Meanwhile, the IEt group displayed enrichment in propanoate metabolism, base excision repair, mismatch repair, and other entries.

### Effects of IEt on Carbohydrate-Active Enzymes

The collective relative abundances of carbohydrate-active enzymes (CAZymes) were determined for each group based on the Carbohydrate-Active enzymes (CAZy) database (Fig. S2A). After radiotherapy, there was a significant increase in the total abundance of CAZymes, which was notably reversed by IEt. Specifically, radiotherapy induced a substantial elevation in the abundance of glycoside hydrolases (GH), glycosyl transferases (GT), carbohydrate-binding modules (CBM), polysaccharide lyases (PL), and carbohydrate esterases (CE). In contrast, IEt resulted in a reduction in the abundance of GH, GT, CBM, and CE (Fig. S2B). The analysis of the relative abundance of CAZymes at level 2 (Fig. S2C) indicated that radiotherapy significantly increased the abundance of CAZymes such as GH10, GT106, and CBM6, all of which were reduced by IEt. Conversely, radiotherapy significantly decreased the abundance of CAZymes such as GH18, CE12, and GT32, which were significantly increased by IEt.

### Effects of IEt on Metabolites

**Fig. 6:**
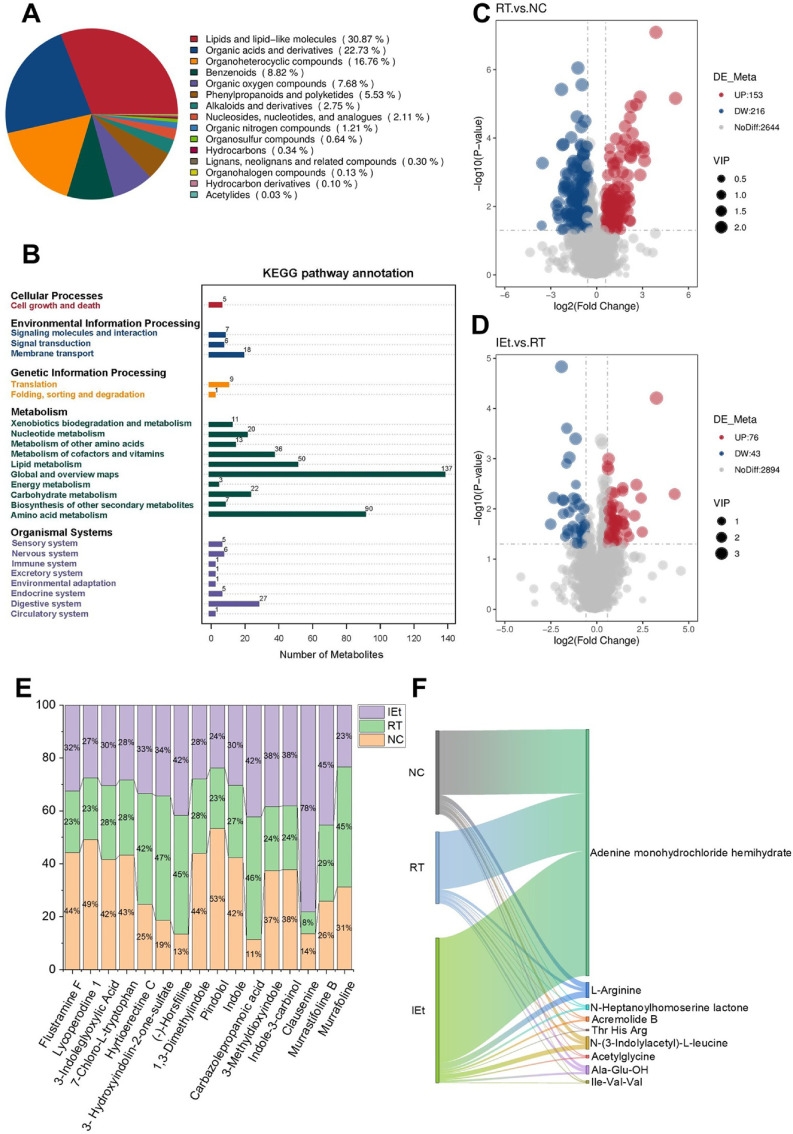
Analysis of metabolite. (**A**) Pie chart of metabolite classification at level 1; (**B**) Statistical diagram of KEGG annotation; (**C-D**) Differential metabolites volcano plot of (**C**) RT vs NC group and (**D**) IEt vs RT group; (**E**) Changes of indole and derivatives; (**F**) Sankey diagram of organic acids and derivatives. NC, the control group; RT, the radiotherapy group; IEt, IEt administration + radiotherapy group.

A total of 3,013 metabolites were detected, with lipids and lipid-like molecules accounted for the highest proportion (30.87%), followed by organic acids and derivatives (22.73%), and organoheterocyclic compounds, which accounted for 16.76% (Fig. 6A). The statistical diagram of KEGG annotation showed the number of metabolites annotated to each secondary category under the primary pathway classification (Fig. 6B). The results showed that at level 1, the item of metabolism was annotated with the most metabolites, totaling 389. Among these, 137 metabolites were annotated in global and overview maps at level 2. This was followed by amino acid metabolism, which was annotated with 90 metabolites. Analysis of the volcano plot (Fig. 6C and 6D) showed that 153 metabolites were significantly increased and 216 metabolites were significantly decreased in the RT group compared to the NC group. Compared to the RT group, 76 metabolites were significantly increased, and 43 metabolites were significantly decreased in abundance in the IEt group. For example, the t-test results showed that the level of indole-3-carbinol decreased by 36.30% in the RT group compared to the NC group (*p* = 0.0550), while it increased by 57.77% in the IEt group compared to the RT group (*p* = 0.0064). Additionally, indole and its derivatives were significantly altered (Fig. 6E). Compared to the NC group, the abundance of indole and its derivatives including flustramine F, lycoperodine 1,3-indoleglyoxylic acid, 7-chloro-L-tryptophan, 1,3-dimethylindole, pindolol, and indole, significantly decreased, while the abundance of hyrtioerectine C, 3-hydroxyindolin-2-one-sulfate, (-)-horsfiline, and carbazolepropanoic acid increased significantly in the RT group. Compared to the RT group, the abundance of 3-methyldioxyindole, indole-3-carbinol, clausenine, and murrastifoline B was significantly upregulated and murrafoline was significantly downregulated in the IEt group. The changes in organic acids and derivatives were shown in the Sankey diagram (Fig. 6F).

**Fig. 7:**
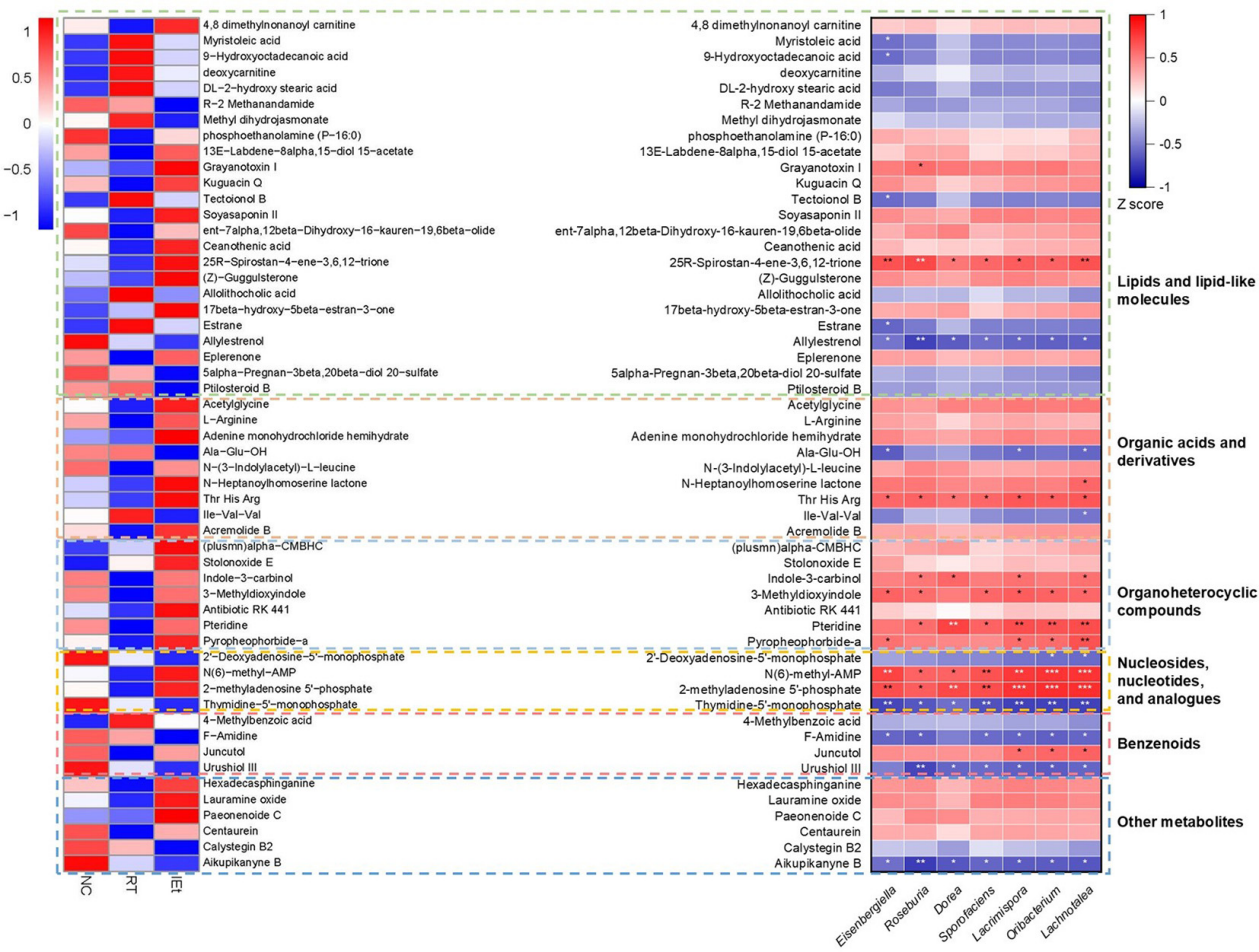
Differential metabolite analysis. Heat map of differential metabolites (left) and correlation analysis between differential metabolites and significantly changed genera belonging to Lachnospiraceae (right). *p < 0.05, **p < 0.01, ***p < 0.001. NC, the control group; RT, the radiotherapy group; IEt, IEt administration + radiotherapy group.

In addition, we analyzed the relative abundance of differential metabolites and their correlation with significantly changed genera belonging to the Lachnospiraceae family (Fig. 7). Spearman correlation analysis showed that the metabolites with anti-inflammatory and anti-cancer effects, including indole-3-carbinol, pteridine, and pyropheophorbide-a, which were significantly upregulated by IEt, were significantly positively correlated with genera of Lachnospiraceae. Specifically, indole-3-carbinol was significantly positively correlated with genera *Roseburia*, *Dorea*, *Lacrimispora*, and *Lachnotalea*; pteridine exhibited a significant positive correlation with the genera *Roseburia*, *Dorea*, *Sporofaciens*, *Lacrimispora*, *Oribacterium*, and *Lachnotalea*; and a positive correlation was observed between pyropheophorbide-a and the genera *Eisenbergiella*, *Lacrimispora*, *Oribacterium*, and *Lachnotalea*.

## Discussion

In this study, a mouse model of radiation-induced enteritis was used to investigate the effect of IEt on radiation enteritis. Analysis of intestinal microbiome composition exhibited significant differences among the three groups, suggesting that radiotherapy and IEt intervention significantly altered the composition and structure of the intestinal microbiome. At the family level, radiotherapy significantly downregulated the abundance of family Lachnospiraceae, which was significantly upregulated by IEt. Lachnospiraceae is a family of anaerobic bacteria in the class Clostridia with potential to advance the intestinal therapeutics. Members of this taxonomic group can ferment dietary fiber to promote healthy gut and immune function [25]. Previous studies have linked Lachnospiraceae to inflammatory diseases in the host. Decreased Lachnospiraceae abundance have been documented in patients with ulcerative colitis (UC) compared to healthy human controls [26]. Another study reported significantly lower levels of Lachnospiraceae taxa, including *Roseburia* and *Coprococcus* in ileal Crohn's disease (CD) patients compared to healthy human controls [27]. These findings are consistent with our results showing that mice with radiation-induced enteritis have a lower abundance of Lachnospiraceae.

IEt may play a radioprotective role by reducing radiation enteritis, which is mediated by family Lachnospiraceae. Lachnospiraceae have been shown to alleviate mucosal inflammation and reduce intestinal radiation injury in mice [28]. In addition, an enrichment of Lachnospiraceae in "elite survival mice" (mice that recovered and survived for a long time after a high dose of whole-body radiation), and subsequent causal experiments showed that the survival rate and clinical score of mice given Lachnospiraceae were greatly improved [11]. Additionally, the radioprotective metabolite mediators were identified as SCFAs and tryptophan metabolites. Treatment of irradiated mice with SCFAs and tryptophan metabolites significantly improved survival and reduced clinical scores [11]. To validate the effects of SCFAs in microbiome-mediated radioprotection, mice were treated with the SCFA producer of Lachnospiraceae strains, and the results revealed that high-producing SCFAs strains ensured complete protection against radiation for all mice, whereas low-producing SCFAs strains provided only 50%protection [11]. Thus, IEt can increase the abundance of Lachnospiraceae to alleviate radiation enteritis and play a radioprotective role in irradiated mice.

IEt reduced the abundance of genera belonging to family Lachnospiraceae including genus *Roseburia*. *Roseburia* has been identified for its role in regulating immune responses and decrease inflammation. *Roseburia* is a beneficial gut bacterium that plays a critical role in protecting against the onset of inflammatory bowel disease by promoting a healthy gut environment and potentially alleviating associated inflammation [29]. *Roseburia* could reduce inflammation through the suppression of the TLR-NF-κB pathway in HT-29 and Caco-2 cells [30]. Additionally, *R. intestinalis* supernatant inhibited colitis, altered inflammatory macrophage activity and pro-inflammatory cytokines, and inhibited Th17 cell differentiation in DSS- and TNBS (2,4,6-trinitrobenzenesulfonic acid solution)-induced IBD mouse models. SCFAs, including butyrate, were subsequently detected in *R. intestinalis* supernatants, which can exert an anti-inflammatory effect [31]. Administration of *R. intestinalis* significantly decreased IL-17 expression and increased the Treg ratio in mice with TNBS-induced colitis [32]. Moreover, the therapeutic application of *R. intestinalis* significantly improved DSS-induced colitis in mice regarding clinical symptoms, histological inflammation, and immune response by regulating Treg/Th17 cell balance and intestinal barrier integrity [33]. Th17 cells are critical members in mediating immune responses of adaptive immunity. In humans and mice, gut is a main site where Th17 cells reside. Radiation can induce the generation and accumulation of Th17 cells in the gut, with IL-17A primarily produced by these cells [34]. In clinical studies, the abundance of *Roseburia* has been found to be reduced in pediatric patients with UC [35] and in those with Crohn's disease [36]. Furthermore, researches indicated that *Roseburia* can enhance the sensitivity of colorectal cancer patients to radiotherapy by generating butyrate [37]. Additionally, *Roseburia* can suppress intestinal inflammation by producing butyrate in patients with colorectal cancer [38]. All these studies demonstrated the beneficial effects of genus *Roseburia* on intestinal inflammation-related diseases. In our study, we found that radiotherapy significantly reduced the abundance of genus *Roseburia* and upregulated the level of pro-inflammatory factor IL-17A, whereas IEt significantly increased *Roseburia* abundance and downregulated IL-17A levels, with a significant negative correlation observed between *Roseburia* and IL-17A. Thus, combined with the findings regarding *Roseburia*’s role in reducing inflammation, IEt may alleviate radiation enteritis through the genus *Roseburia*-IL-17A axis.

Subsequent analysis of the metabolic function of gut microbiota revealed that many metabolites, including indole-3-carbinol, pteridine, and pyropheophorbide-a, were significantly upregulated in irradiated mice treated with IEt. Notably, indole-3-carbinol is a bioactive substance with anti-inflammatory properties. An *in vitro* study showed that indole-3-carbinol ameliorated necroptosis and inflammation in NCM460 cells (a normal human colorectal cell line) [39]. In addition, multiple *in vivo* studies assessed the anti-inflammatory activity of indole-3-carbinol. A study in C57BL/6 mice treated with indole-3-carbinol for TNBS-induced colitis showed that indole-3-carbinol reduced the severity of the disease in female mice, as indicated by reduced weight loss and clinical symptom severity [40]. Likewise, indole-3-carbinol attenuated necroptosis and inflammation in intestinal epithelial cells (IECs) by activating aryl hydrocarbon receptor (AHR), which played a protective role in a mouse model of DSS-induced ulcerative colitis [39]. Furthermore, indole-3-carbinol has been reported to alleviate symptoms through stimulating IL-22 expression in a murine colitis model, which resulted in the generation of anti-inflammatory butyrate by the intestinal microbiome [41]. Moreover, another experiment revealed that indole-3-carbinol reduced the mRNA expression levels of pro-inflammatory cytokines, including IL-17A, IL-6, IL-1β, TNF-α and IFN-γ, in colon tissues of mice [42]. In our study, indole-3-carbinol levels significantly increased after intragastric administration of IEt, and a significant positive correlation was observed between indole-3-carbinol levels and genus *Roseburia*. Hence, combined with the anti-inflammatory effects of indole-3-carbinol, IEt may alleviate radiation enteritis through the genus *Roseburia*-indole-3-carbinol axis.

Interestingly, indole-3-carbinol has also been reported as a promising cancer preventive agent [43]. *In vitro* experiments showed that indole-3-carbinol, as an inhibitor of NF-κB and Akt activation, triggered G1 cell cycle arrest and apoptosis in prostate cancer cells [44]. In addition, several *in vivo* experiments have confirmed the anti-cancer effects of indole-3-carbinol. Indole-3-carbinol inhibited tumor growth in nasopharyngeal carcinoma in mice [45]. Indole-3-carbinol inhibited the development of cervical cancer in mice by upregulating *PTEN* expression [46]. In short, IEt can exert anti-inflammatory and radioprotective effects by regulating the abundance of Lachnospiraceae, and it also plays an anti-tumor role mediated by indole-3-carbinol during radiotherapy in mice.

Although this study identified IEt as a promising radioprotective metabolite, several limitations require consideration. Due to the lack of mechanistic studies involving bacterial depletion or pathway inhibition, causal claims regarding the *Roseburia*-indole-3-carbinol and *Roseburia*-IL-17A axes have not been verified. Furthermore, the scope of inflammatory profiling was restricted to select cytokines, omitting broader immune cell or spatial tissue analyses. To address these gaps, subsequent investigations will prioritize large animal validation in porcine radiation models – a critical step for clinical translation – alongside mechanistic studies in gnotobiotic mice and AHR-knockout systems to dissect microbial-metabolite-immune crosstalk. Targeted metabolomics will further resolve SCFA dynamics and other bioactive metabolites, ensuring comprehensive mechanistic elucidation.

**Fig. 8:**
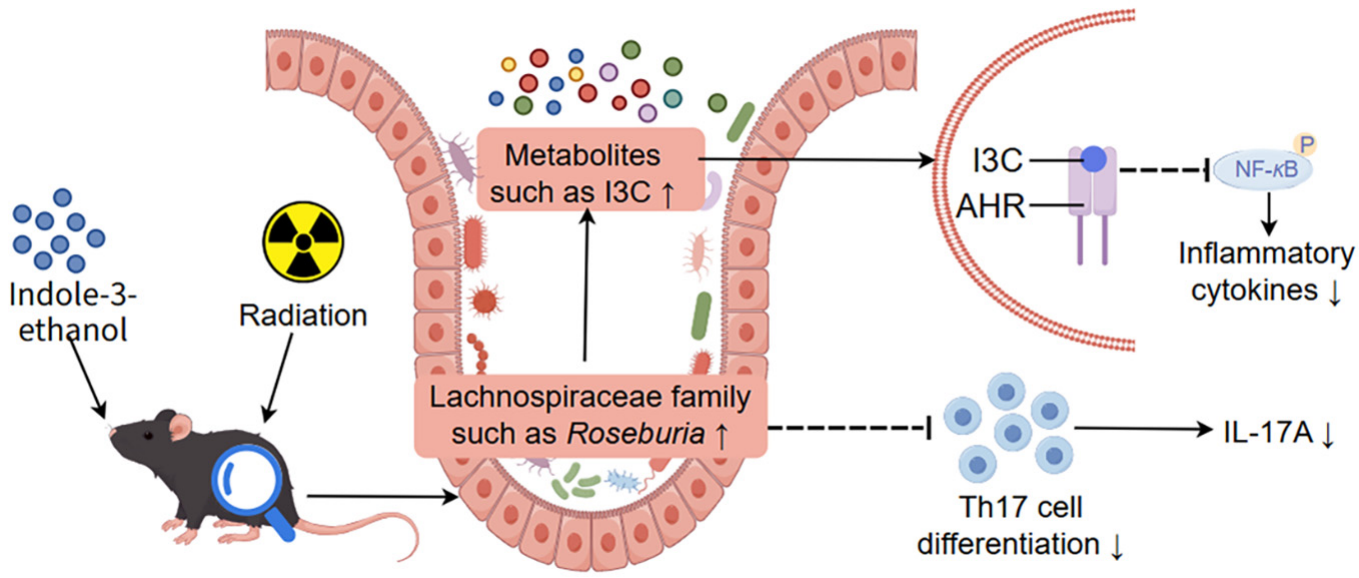
Indole-3-ethanol alleviated radiation-induced enteritis through *Roseburia*-indole-3-carbinol and *Roseburia*-IL-17A axes in mice. I3C, indole-3-carbinol; Th17, T helper cell 17; IL-17A, interleukin-17A; AHR, aryl hydrocarbon receptor; NF-κB, nuclear factor kappa-B; P, phosphorylation.

In conclusion, IEt decreased the levels of the pro-inflammatory factor, such as IL-17A, and increased the abundance of anti-inflammatory Lachnospiraceae, such as *Roseburia*, as well as the levels of the anti-inflammatory metabolite indole-3-carbinol. Additionally, *Roseburia* showed a significant positive correlation with indole-3-carbinol and a significant negative correlation with IL-17A. Therefore, IEt may alleviate radiation enteritis through genus *Roseburia*-indole-3-carbinol and *Roseburia*-IL-17A axes (Fig. 8). This study identified a new tryptophan-derived microbial metabolite, IEt, as a protective agent against radiation enteritis, providing theoretical support for the prevention of radiation enteritis.

## Supplemental Materials

Supplementary data for this paper are available on-line only at http://jmb.or.kr.



## References

[1] Bray F, Laversanne M, Sung H, Ferlay J, Siegel RL, Soerjomataram I (2024). Global cancer statistics 2022: GLOBOCAN estimates of incidence and mortality worldwide for 36 cancers in 185 countries. CA Cancer J. Clin..

[2] Hauer-Jensen M, Denham JW, Andreyev HJ (2014). Radiation enteropathy--pathogenesis, treatment and prevention. Nat. Rev. Gastroenterol. Hepatol..

[3] Gong L, Zhang Y, Liu C, Zhang M, Han S (2021). Application of radiosensitizers in cancer radiotherapy. Int. J. Nanomedicine.

[4] Jian Y, Zhang D, Liu M, Wang Y, Xu ZX (2021). The impact of gut microbiota on radiation-induced enteritis. Front. Cell. Infect. Microbiol..

[5] Yi Y, Lu W, Shen L, Wu Y, Zhang Z (2023). The gut microbiota as a booster for radiotherapy: novel insights into radio-protection and radiation injury. Exp. Hematol. Oncol..

[6] Andreyev J (2005). Gastrointestinal complications of pelvic radiotherapy: are they of any importance?. Gut.

[7] Moraitis I, Guiu J, Rubert J (2023). Gut microbiota controlling radiation-induced enteritis and intestinal regeneration. Trends Endocrinol. Metab..

[8] Shadad AK, Sullivan FJ, Martin JD, Egan LJ (2013). Gastrointestinal radiation injury: symptoms, risk factors and mechanisms. World J. Gastroenterol..

[9] Ghimire S, Cady NM, Lehman P, Peterson SR, Shahi SK, Rashid F (2022). Dietary isoflavones alter gut microbiota and lipopolysaccharide biosynthesis to reduce inflammation. Gut Microbes.

[10] Patterson GT, Osorio EY, Peniche A, Dann SM, Cordova E, Preidis GA (2022). Pathologic inflammation in malnutrition is driven by proinflammatory intestinal microbiota, large intestine barrier dysfunction, and translocation of bacterial lipopolysaccharide. Front. Immunol..

[11] Guo H, Chou WC, Lai Y, Liang K, Tam JW, Brickey WJ (2020). Multi-omics analyses of radiation survivors identify radioprotective microbes and metabolites. Science.

[12] Gerassy-Vainberg S, Blatt A, Danin-Poleg Y, Gershovich K, Sabo E, Nevelsky A (2018). Radiation induces proinflammatory dysbiosis: transmission of inflammatory susceptibility by host cytokine induction. Gut.

[13] Xie LW, Cai S, Lu HY, Tang FL, Zhu RQ, Tian Y (2024). Microbiota-derived I3A protects the intestine against radiation injury by activating AhR/IL-10/Wnt signaling and enhancing the abundance of probiotics. Gut Microbes.

[14] Xiao HW, Cui M, Li Y, Dong JL, Zhang SQ, Zhu CC (2020). Gut microbiota-derived indole 3-propionic acid protects against radiation toxicity via retaining acyl-CoA-binding protein. Microbiome.

[15] Rooks MG, Garrett WS (2016). Gut microbiota, metabolites and host immunity. Nat. Rev. Immunol..

[16] Donia MS, Fischbach MA (2015). Small molecules from the human microbiota. Science.

[17] Scott SA, Fu J, Chang PV (2020). Microbial tryptophan metabolites regulate gut barrier function via the aryl hydrocarbon receptor. Proc. Natl. Acad. Sci. USA.

[18] Li Z, Gao Y, Du L, Yuan Y, Huang W, Fu X (2022). Anti-inflammatory and anti-apoptotic effects of Shaoyao decoction on X-ray radiation-induced enteritis of C57BL/6 mice. J. Ethnopharmacol..

[19] Zhang Y, Qin S, Song Y, Yuan J, Hu S, Chen M (2022). Alginate oligosaccharide alleviated cisplatin-induced kidney oxidative stress via *Lactobacillus* genus-FAHFAs-Nrf2 axis in mice. Front. Immunol..

[20] Hou Y, Wang X, Chen X, Zhang J, Ai X, Liang Y (2019). Establishment and evaluation of a simulated high-altitude hypoxic brain injury model in SD rats. Mol. Med. Rep..

[21] Want EJ, Masson P, Michopoulos F, Wilson ID, Theodoridis G, Plumb RS (2013). Global metabolic profiling of animal and human tissues via UPLC-MS. Nat. Protoc..

[22] Karlsson FH, Tremaroli V, Nookaew I, Bergstrom G, Behre CJ, Fagerberg B (2013). Gut metagenome in European women with normal, impaired and diabetic glucose control. Nature.

[23] Karlsson FH, Fak F, Nookaew I, Tremaroli V, Fagerberg B, Petranovic D (2012). Symptomatic atherosclerosis is associated with an altered gut metagenome. Nat. Commun..

[24] Nielsen HB, Almeida M, Juncker AS, Rasmussen S, Li J, Sunagawa S (2014). Identification and assembly of genomes and genetic elements in complex metagenomic samples without using reference genomes. Nat. Biotechnol..

[25] Zaplana T, Miele S, Tolonen AC (2023). Lachnospiraceae are emerging industrial biocatalysts and biotherapeutics. Front. Bioeng. Biotechnol..

[26] Frank DN, St Amand AL, Feldman RA, Boedeker EC, Harpaz N, Pace NR (2007). Molecular-phylogenetic characterization of microbial community imbalances in human inflammatory bowel diseases. Proc. Natl. Acad. Sci. USA.

[27] Baumgart M, Dogan B, Rishniw M, Weitzman G, Bosworth B, Yantiss R (2007). Culture independent analysis of ileal mucosa reveals a selective increase in invasive *Escherichia coli* of novel phylogeny relative to depletion of *Clostridiales* in Crohn's disease involving the ileum. ISME J..

[28] Liu L, Lu X, Li S, Zhang P (2024). Polysaccharides isolated from *Hibiscus sabdariffa* L. mitigate intestinal radiation injury through relieving mucosal inflammation and reshaping gut microbiota in mice. J. Funct. Foods.

[29] Nie K, Ma K, Luo W, Shen Z, Yang Z, Xiao M (2021). *Roseburia intestinalis*: a beneficial gut organism from the discoveries in genus and species. Front. Cell. Infect. Microbiol..

[30] Lakhdari O, Tap J, Béguet-Crespel F, Le Roux K, de Wouters T, Cultrone A (2011). Identification of NF-κB modulation capabilities within human intestinal commensal bacteria. J. Biomed. Biotechnol..

[31] Luo W, Shen Z, Deng M, Li X, Tan B, Xiao M (2019). *Roseburia intestinalis* supernatant ameliorates colitis induced in mice by regulating the immune response. Mol. Med. Rep..

[32] Zhu C, Song K, Shen Z, Quan Y, Tan B, Luo W (2018). *Roseburia intestinalis* inhibits interleukin-17 excretion and promotes regulatory T cells differentiation in colitis. Mol. Med. Rep..

[33] Mohebali N, Weigel M, Hain T, Sutel M, Bull J, Kreikemeyer B (2023). *Faecalibacterium prausnitzii*, *Bacteroides faecis* and *Roseburia intestinalis* attenuate clinical symptoms of experimental colitis by regulating Treg/Th17 cell balance and intestinal barrier integrity. Biomed. Pharmacother..

[34] Shao L, Li M, Zhang B, Chang P (2020). Bacterial dysbiosis incites Th17 cell revolt in irradiated gut. Biomed. Pharmacother..

[35] Shah R, Cope JL, Nagy-Szakal D, Dowd S, Versalovic J, Hollister EB (2016). Composition and function of the pediatric colonic mucosal microbiome in untreated patients with ulcerative colitis. Gut Microbes.

[36] Imhann F, Vich Vila A, Bonder MJ, Fu J, Gevers D, Visschedijk MC (2018). Interplay of host genetics and gut microbiota underlying the onset and clinical presentation of inflammatory bowel disease. Gut.

[37] Dong J, Wang B, Xiao Y, Liu J, Wang Q, Xiao H (2024). *Roseburia intestinalis* sensitizes colorectal cancer to radiotherapy through the butyrate/OR51E1/RALB axis. Cell Rep..

[38] Kang X, Liu C, Ding Y, Ni Y, Ji F, Lau HCH (2023). *Roseburia intestinalis* generated butyrate boosts anti-PD-1 efficacy in colorectal cancer by activating cytotoxic CD8^+^ T cells. Gut.

[39] Peng C, Wu C, Xu X, Pan L, Lou Z, Zhao Y (2021). Indole-3-carbinol ameliorates necroptosis and inflammation of intestinal epithelial cells in mice with ulcerative colitis by activating aryl hydrocarbon receptor. Exp. Cell Res..

[40] Benson JM, Beamer CA, Seaver BP, Shepherd DM (2012). Indole-3-carbinol exerts sex-specific effects in murine colitis. Eur. J. Inflamm..

[41] Busbee PB, Menzel L, Alrafas HR, Dopkins N, Becker W, Miranda K (2020). Indole-3-carbinol prevents colitis and associated microbial dysbiosis in an IL-22-dependent manner. JCI Insight.

[42] Wu Y, Wang J, He Q, Yu L, Pham Q, Cheung L (2020). Dietary indole-3-carbinol alleviated spleen enlargement, enhanced IgG response in C3H/HeN mice infected with *Citrobacter rodentium*. Nutrients.

[43] Karimabad MN, Mohamadi M, Torabizadeh SA, Hassanshahi G (2023). Indole-3-carbinol (I3C) as leukemia therapeutic agents: review. Mini Rev. Med. Chem..

[44] Sarkar FH, Li Y (2004). Indole-3-carbinol and prostate cancer. J. Nutr..

[45] Zhu W, Li W, Yang G, Zhang Q, Li M, Yang X (2010). Indole-3-carbinol inhibits nasopharyngeal carcinoma. Int. J. Toxicol..

[46] Qi M, Anderson AE, Chen DZ, Sun S, Auborn KJ (2005). Indole-3-carbinol prevents *PTEN* loss in cervical cancer *in vivo*. Mol. Med..

