# Supplementary material for: Metagenomics-Metabolomics Reveals the Alleviation of Indole-3-Ethanol on Radiation-Induced Enteritis in Mice
Source: J Microbiol Biotechnol. 2025 Jul 18;35:e2502037. doi: 10.4014/jmb.2502.02037 (PMC12324999; doi:10.4014/jmb.2502.02037)
Supplement: Supplementary file 1 [file jmb-35-e2502037-supple.pdf]

J. Microbiol. Biotechnol. 2025. 35: e2502037
https://doi.org/10.4014/jmb.2502.02037

Supplementary Table and Figures

Table S1. The component of standard chow.

Component	Content (g/kg)
Water	95.0
Crude protein	220
Crude fat	50
Crude fiber	30
Crude ash	60
Calcium	12.5
Phosphorus	7.5
Lysine	15.9
Methionine+Cystine	9.9

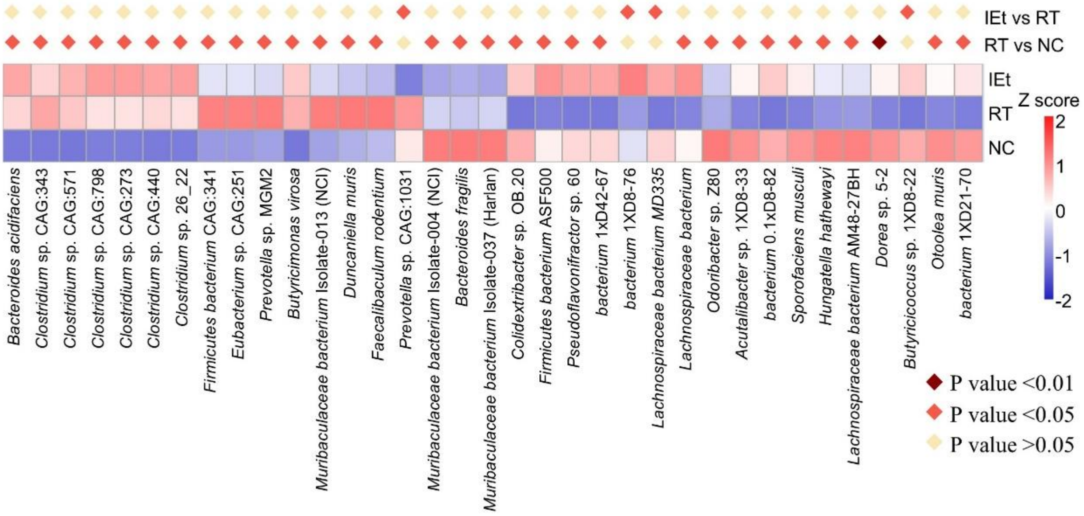

Fig. S1. Changes of the gut microbiota at species level. NC, the control group; RT, the radiotherapy group; IEt, IEt administration + radiotherapy group.

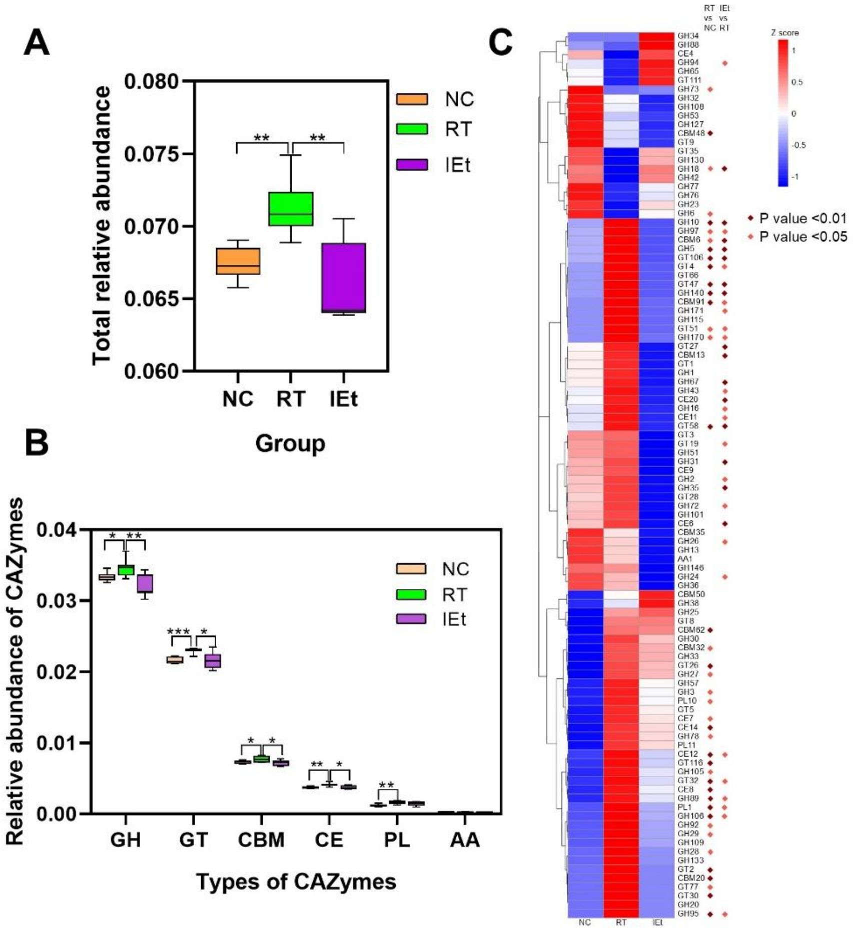

Fig. S2. Analysis of CAZymes. (**A**) Total relative abundance of the three groups of CAZymes; (**B**) The relative abundance of CAZymes at level 1; (**C**) The relative abundance of CAZymes at level 2. * $p<0.05$, ** $p<0.01$, *** $p<0.001$. GH, glycoside hydrolases; GT, glycosyl transferases; CBM, carbohydrate-binding modules; CE, carbohydrate esterases; PL, polysaccharide lyases; AA, auxiliary activities; NC, the control group; RT, the radiotherapy group; IEt, IEt administration + radiotherapy group.
